# Myopia Control with a Novel Peripheral Gradient Soft Lens and Orthokeratology: A 2-Year Clinical Trial

**DOI:** 10.1155/2015/507572

**Published:** 2015-10-28

**Authors:** Jaime Pauné, Hari Morales, Jesús Armengol, Lluisa Quevedo, Miguel Faria-Ribeiro, José M. González-Méijome

**Affiliations:** ^1^Centro Médico Teknon, 08022 Barcelona, Spain; ^2^Departament d'Òptica i Optometria, Universitat Politècnica de Catalunya-BarcelonaTech (UPC), 08222 Terrassa, Spain; ^3^Clinical & Experimental Optometry Research Lab (CEORLab), Center of Physics, University of Minho, 4710-057 Braga, Portugal

## Abstract

*Objective.* To evaluate the degree of axial elongation with soft radial refractive gradient (SRRG) contact lenses, orthokeratology (OK), and single vision (SV) spectacle lenses (control) during a period of 1 year before treatment and 2 years after treatment. *Methods.* This was a prospective, longitudinal, nonrandomized study. The study groups consisted of 30, 29, and 41 children, respectively. The axial length (AL) was measured during 2 years after recruitment and lens fitting. *Results.* The baseline refractive sphere was correlated significantly (Spearman's Rho (*ρ*) correlation = 0.542; *P* < 0.0001) with the amount of myopia progression before baseline. After 2 years, the mean myopia progression values for the SRRG, OK, and SV groups were −0.56 ± 0.51, −0.32 ± 0.53, and −0.98 ± 0.58 diopter, respectively. The results represent reductions in myopic progression of 43% and 67% for the SRRG and OK groups, respectively, compared to the SV group. The AL increased 27% and 38% less in the SRRG and OK groups, respectively compared with the SV group at the 2-year visit (*P* < 0.05). Axial elongation was not significantly different between SRRG and OK (*P* = 0.430). *Conclusion.* The SRRG lens significantly decreased AL elongation compared to the SV control group. The SRRG lens was similarly effective to OK in preventing myopia progression in myopic children and adolescent.

## 1. Introduction 

Myopia is associated with ocular complications that can lead to permanent vision loss [1]. This is especially true in relation to the amount of refractive error that is related to an increased risk of retinal detachment, glaucoma, cataract, and chorioretinal degeneration as the leading causes of permanent visual impairment [2].

Studies of the emmetropization mechanism in animals have suggested that treatments that consider the peripheral retina may be much more effective than others [3]. In particular, experiments in monkeys have indicated that visual signals from the peripheral retina are essential for several aspects of regulation of vision-dependent ocular growth [4]. Moreover, recent studies have shown that inducing a multifocal image on the eye and moving the image forward at the peripheral retina, leaving it myopically defocused, generates a visual stimulus to slow ocular growth [5, 6].

Several strategies using optical devices have been developed to reduce myopic progression. Undercorrection actually accelerates myopia progression [7]. Progressive addition lenses have a significant but clinically small effect [8, 9] that is potentially related to changes in defocus of superior retinal images [10]. Bifocal executive lenses have shown significant and promising results especially in esophoric children and those with rapid progression [11]. An experimental spectacle lens designed to decrease peripheral hyperopia reduced myopic progression by 30% over a 12-month period [12]. Rigid gas permeable contact lenses also have a small effect that can be confounded by the keratometric changes [13].

Orthokeratology (OK) slows axial elongation of the eye by about 50% [14] and is the current technique with the most consistent results, except in low myopes with less than −2.00 diopters (D), in whom this therapy seems to have less benefit according to one study [15]. This finding is likely related to the fact that the change in corneal curvature is lower in these patients and results in less peripheral myopization. The induced peripheral myopia regarding the spherical equivalent has an almost 1 : 1 relationship with the amount of baseline spherical equivalent refraction to be corrected [16]. Although some investigators have attempted to overcome this limitation, they were unsuccessful [17]. For myopia exceeding −6.00 D, the treatment is performed “off-label” in some countries, and usually the fit is more challenging. In addition, due to particular ocular characteristics, not all patients are candidates to orthokeratology treatment.

Soft multifocal contact lenses are advantageous compared to ophthalmic lenses because they move with the eye and thus the optical correction remains centered for all gaze positions. Previous studies of multifocal contact lenses have reported reductions in myopic progression ranging from 30% to 50% and about 30% in axial length (AL) depending on lens design [18–22]. Generally, all experimental lenses tested were designed with a central optic zone intended for distance vision and surrounded by one or more rings with plus addition powers. Further, previous works have shown reductions in the ocular growth rate in eyes fitted with multifocal soft contact lenses compared with monofocal contact lenses or spectacle controls. In the current study, we evaluated an experimental SRRG contact lens designed to correct the central refraction and simultaneously produce constant peripheral myopization defocus that increased gradually from the central optic axis toward the periphery. The details of the optical design were reported elsewhere [23, 24].

Since previous studies have demonstrated that peripheral refraction can be changed towards a relative peripheral myopic condition using orthokeratology [16, 17] or peripheral gradient contact lenses [18, 23, 24], the primary goal of the current study was to investigate the myopic control effect of the SRRG experimental lens. A second goal was to compare its efficacy with that of OK, the current golden standard in myopia control with contact lenses. To the best of our knowledge, this is the first controlled clinical trial to provide information about myopic progression 12 months before entry into the study, compare the efficacy of two treatments with different contact lenses, and present insight into the potential causes of myopia control by analyzing the peripheral refraction.

## 2. Materials and Methods

### 2.1. Participants

Three study groups were recruited for this trial from 127 Caucasian clinical patients at the Centro Médico Teknon. The participants were recruited from May 2011 to September 2012. The Ethics Committee for Clinical Research of Centro Médico Teknon approved the study protocol, which adhered to the tenets of the Declaration of Helsinki. All parents provided signed consent for their children to participate. The inclusion criteria were ages from 9 to 16 years at the baseline visit, spherical refractive error between −0.75 and −7.00 D, less than −1.25 D of astigmatism measured by cycloplegic autorefraction, a best spectacle-corrected visual acuity of 20/20 or better in each eye, and an increase in myopia of at least −0.30 D/year over the previous 12 months before entering the study. Cycloplegic autorefraction was obtained before the study entry by the same clinician using the same instrument and measurement protocol. A regression line was fitted to the data to calculate the slope of the annual increase in myopia. The maximal annual myopia refractive increase of the participants was −1.29 D. The exclusion criteria were anisometropia exceeding 1.00 D, strabismus, and any systemic or ocular disease that may affect ocular growth or contact lens wear.

Of the 127 subjects recruited, 27 were excluded for not having the minimum annual myopia progression of −0.30 D/year. The final study groups included 30 children in the soft SRRG group, 29 in the OK group, and 41 in the single vision spectacle lenses (SV) group.

### 2.2. Sample size

The sample size was calculated to determine whether the patients in the SRRG and OK groups progressed slower than those in the SV group. The standard deviations (SDs) of the 2-year changes in AL and refractive error were assumed to be 0.15 mm and 0.50 D, respectively. To have 80% power for a significance level of *α* = 0.05 with a confidence level of 95% to detect a difference of 0.15 mm and 0.50 D over 2 years, the minimum numbers of subjects required in each group were 19 and 17, respectively.

### 2.3. Study Design

This was a prospective, longitudinal, nonrandomized study. After receiving an explanation of the study, the parents chose the treatment for their child. The AL and refraction were obtained without correction in all groups every 6 months over 2 years. Soft contact lens wearers were instructed to not wear the lenses for 2 days before the follow-up examinations to avoid any potential corneal warpage that might induce errors in the estimation of the refractive error. The main outcomes of efficacy of myopic control were determined by comparing the differences in the mean changes in AL and spherical equivalent (M) among the three groups after 2 years.

### 2.4. Contact Lenses

The experimental SRRG lens designed to produce peripheral myopic defocus was fitted after a baseline examination that included refraction and corneal measures obtained without refractive correction. The contact lens was made of 2-hydroxy-ethyl methacrylate, a nonionic material, with 38% water content and 12 barrer of oxygen permeability (Dk) (Servilens, Granada, Spain). The central thickness varied with optical power ranging from 0.09 to 0.14 mm. The overall diameter was 14.00 to 15.00 mm. The base curve radius ranged from 8.00 to 8.90 mm and was calculated to be 0.7 mm flatter than the average keratometric radius. Experimental soft lenses have unique central back and front optical zones of 8 mm in diameter, and only the central apical zone had the power required for distance vision. The progressive design provided an increasing add power that reached +2.00 D add plus power, which corresponded to about 35 degrees of retinal eccentricity and achieved about +6.00 D of addition plus power at the edge of the optical zone (4 mm semichord diameter) [23]. Contact lens fitting was performed according to the subjective refraction, corneal curvature, and visible iris diameter. The corneal topography was measured using the Keratron Scout Corneal Topographer (Opticon 2000 SpA, Rome, Italy). Adjustments to the final prescription were based on spherical overrefraction, and a new lens was ordered if discrepancies exceeding ±0.25 D were found. Fitting was assessed for centration and lag on lateral gaze movements using the slit-lamp beam. All lenses were within the desired limits of less than 0.50 mm of movement on blink in upgaze and 1.00 mm lag in lateral gaze. Measurements were obtained without correction in SRRG and SV spectacle control groups.

The OK group was fitted with a Double Reservoir Lens (DRL) (Precilens, Paris, France), previously calculated according to the manufacturer's protocols that considered the topographic values and refraction. All fittings were optimized until centration and the correct refractive outcomes were achieved. DRL lenses are made of a Boston XO2 (hexafocon B) material with an oxygen permeability of 141 barriers, refractive index of 1.424, Rockwell R hardness of 101 units, and wetting angle of 38 degrees measured with the captive bubble method.

### 2.5. Primary Outcomes

The refractive error was measured in 0.01 D steps with cycloplegic autorefraction using the Grand Seiko Autorefractometer/Keratometer WAM-5500 (Grand Seiko Co., Ltd., Hiroshima, Japan), with the same protocol used for all three groups.

Cycloplegia was achieved using two drops of cyclopentolate 1% separated by 10 minutes each. The same examiner performed and averaged five consecutive measurements 30 minutes after the second drop was instilled.

The refraction was adjusted using keratometric changes from baseline to avoid any change in the anterior surface due to a warpage effect from the soft lenses. In the OK group, the refraction was measured over the OK lens during all visits, with the lens centered between blinks, which is achieved considering the diameter and fitting characteristics achieved with the DRL. The keratometry values of the anterior contact lens surface were assessed to assure that no lens flexure occurred.

The AL was measured in 0.01 mm steps under cycloplegia obtained using cyclopentolate hydrochloride 1.0% (Alcon, El Masnou, Spain) and anesthesia using oxybuprocaine hydrochloride 0.4% and tetracaine hydrochloride 0.1% (Alcon) using the OcuScan RxP Ophthalmic Ultrasound System (Alcon, Fort Worth, TX, USA). Ecographic signals were examined for relatively equal lens peaks and well defined retinal peaks. The same experienced optometrist performed 10 consecutive measurements. When poor signals were detected, measures were repeated. The mean axial dimensions were calculated as the mean of the 10 readings.

### 2.6. Other Measurements

The keratometry and corneal eccentricity values were retrieved to analyze the longitudinal corneal changes for the SRRG and SV lens. Corneal pachymetry was performed before biometry using the same instrument (OcuScan RxP) with the appropriate probe. The relative peripheral refractive error (RPRE) was obtained at 30 degrees of the nasal and temporal retinal eccentricities at baseline without and with the SRRG lenses using the Grand Seiko Autorefractometer/Keratometer WAM-5500.

### 2.7. Statistical Analysis

To analyze myopic progression for 1 year before and at 6, 12, and 18 months, and 24 months of follow-up after lens fitting, only the data from children who completed the study were included. At the end of the treatment, 11 participants in the SRRG group were not included primarily because eight were lost to follow-up, two had lens discomfort, and one moved away from the city. In the OK group, one child had peripheral infiltrative keratitis and left the study, three moved, and seven were lost to follow-up. In the SV group, 20 patients were lost to follow-up. All clinical conditions were treated adequately and recovered without visual loss.

**Figure 1:**
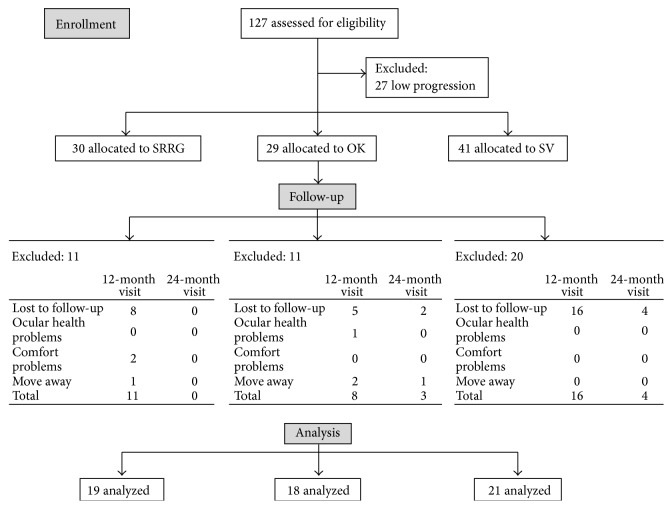
Flow diagram of study progress in the SRRG, OK and SV groups.

At the 2-year visit, 19, 18, and 21 eyes were included in the final analysis in the SRRG, OK, and SV groups, respectively (Figure 1).

All analyses were performed using the SPSS software package version 19 (SPSS Inc., Chicago, IL, USA). The Kolmogorov-Smirnov test was applied to assess the normality of data distribution. Analysis of variance (ANOVA) and the Kruskal-Wallis test were used for comparisons among all groups to assess differences among the SRRG, OK, and SV groups for normally or non-normally distributed variables, respectively. The paired sample test and Wilcoxon signed-ranks test were used for comparisons between two different conditions (SRRG and SV, SRRG and OK, and SV and OK) for normally or non-normally distributed variables, respectively. Spearman's rho (*ρ*) correlation was applied when normality could not be assumed, and the Pearson correlation was used when normal distribution of data was verified to evaluate the relationship between refractive change and AL change and the peripheral refractive error at baseline and myopic change.

To evaluate the biometric changes during the study, we calculated the slope of the linear regression for the changes in each individual in biometric parameters during the 2-year evaluation (anterior chamber depth (ACD); lens thickness (L); vitreous chamber depth (VCD); and AL). We then performed nonparametric Kruskal-Wallis test to evaluate the significance of the intragroup slope. For statistical purposes, *P* < 0.05 was considered significant.

## 3. Results

### 3.1. Baseline Values

Table 1 shows the baseline data. No significant (*P* > 0.05) differences were seen among the groups in gender, age, M refractive error component, keratometry, corneal eccentricity, pachymetry, ACD, lens thickness, vitreous chamber, or AL.

### 3.2. RPRE Related to Myopia Increase

A small but significant correlation was found between the baseline sphere and the amount of M RPRE for the temporal and nasal retina (*ρ* = −0.279; *P* = 0.02; and *ρ* = −0.223; *P* = 0.05, resp.) with higher degrees of myopia also having higher degrees of relative peripheral hyperopia. The baseline refractive sphere was highly correlated with the amount of myopic progression before baseline (*ρ* = 0.542; *P* < 0.0001).

**Figure 2:**
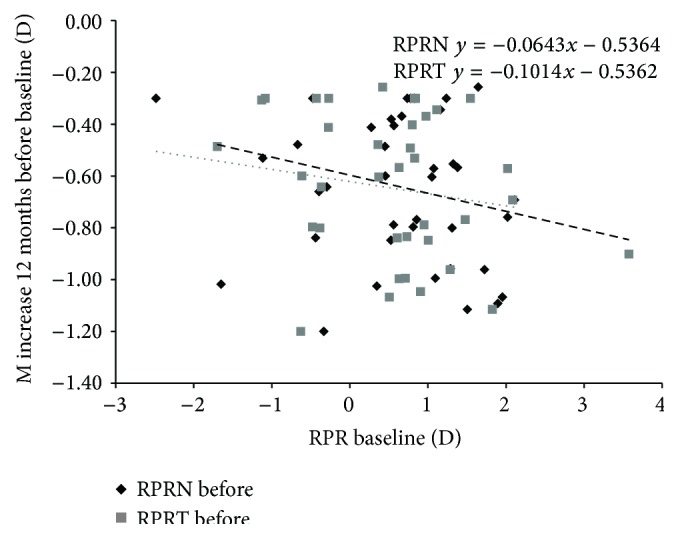
Increase in M from 1 year before treatment against baseline RPRE (M value at the temporal and nasal retina). Correlations were statistically significant for the temporal and nasal RPRE (*P* < 0.01 and *P* < 0.05, resp.). Every 1.00 D of hyperopic RPRE temporal M value is related to a −0.10 D extra increase in annual M value. The dotted line indicates the regression of the RPRE-T (temporal retina) and the dashed line indicates the RPRE-N (nasal retina).

Significant correlations were found between the baseline RPRE-M (RPRE as the spherical equivalent of refraction “M”) at 30 degrees and the rate of myopic progression during 1 year before baseline. The values were significantly correlated at the temporal and nasal retina (*ρ* = −0.295, *P* < 0.01; and *ρ* = −0.254; *P* < 0.05, resp.), suggesting that higher degrees of peripheral hyperopia were associated with greater central progression during 1 year before the trial (Figure 2). No correlations were found between the RPRE-J0 and RPRE-J45 (RPRE as the horizontal and oblique astigmatic components of refraction “J0” and “J45,” resp.), nasal or temporal, refraction components, and the myopic increase during 1 year before the baseline evaluation.

**Figure 3:**
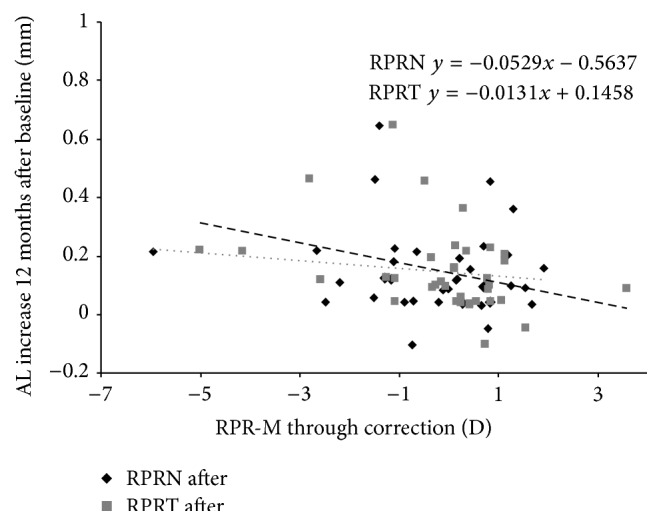
AL increase in relation to the RPRE in the nasal and temporal retina measured through the SRRG (experimental group) and SV (control group). The black diamonds indicate the RPRE-T (temporal retina); the grey squares indicated the RPRE-N (nasal retina); the dotted line indicates the regression of the RPRE-T; and the dashed line indicates the RPRE-N.

The RPRE also was measured at 30 degrees of the nasal and temporal retinal eccentricities at baseline through the experimental lens. No correlation was found between the myopic RPRE-M measured with the SRRG at the nasal (*P* = 0.270) and temporal (*P* = 0.940) eccentricities and the amount of refractive or AL change for the first year in the SRRG group. Moreover, the baseline RPRE (M, J0, and J45) was not correlated significantly with the myopia refractive change at 1 year in this group. However, the RPRE-M at the temporal and nasal retina measured in all participants through the corresponding visual correction (soft experimental lenses and glasses) was correlated with the increase in the AL for the first year of treatment (RPRE-N, *ρ* = −0.400; *P* < 0.001 and RPRE-T, *ρ* = −0.241; *P* < 0.05) (Figure 3).

### 3.3. Spherical Equivalent: Changes at 6, 12, 18 Months and 2 Years

**Figure 4:**
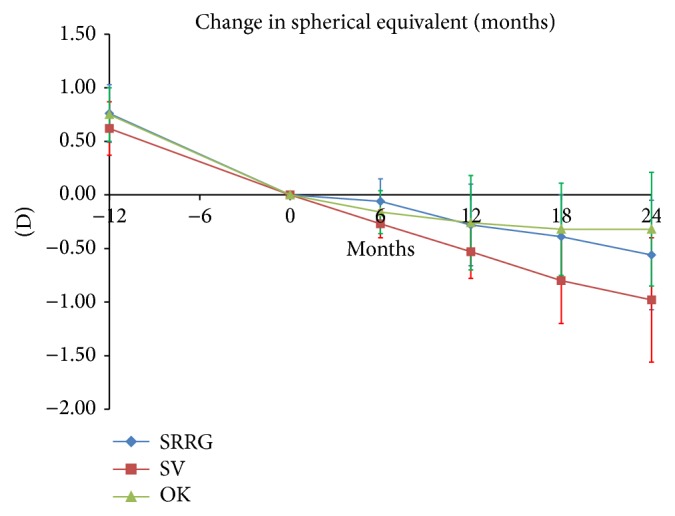
Mean and SD of myopic progression (spherical equivalent refraction).

All groups had similar rates of progression over the year before recruitment. The mean values were, −0.76 ± 0.27 D/year, −0.75 ± 0.25 D/year, and −0.62 ± 0.25 D/year for the SRRG, OK and SV groups, respectively (*P* = 0.156). The SV group had higher changes in spherical equivalent (M) than the SRRG and OK groups during the full length of the study. After 2 years, the mean rates of myopic progression for the SRRG, OK, and SV groups were −0.56 ± 0.51 D, −0.32 ± 0.53 D, and −0.98 ± 0.58 D, respectively. This represents a reduction in myopic progression of 43% and 67% for the SRRG and OK groups, respectively, compared to the SV group. The difference, although larger in the OK group, did not differ significantly (*P* = 0.163) from the SRRG group.  Table 2 and Figure 4 show the mean myopic progression for the three groups at each 6-month interval for the 2 years of the study.

### 3.4. The Biometric Changes at 6, 12, 18 Months and 2 Years

**Figure 5:**
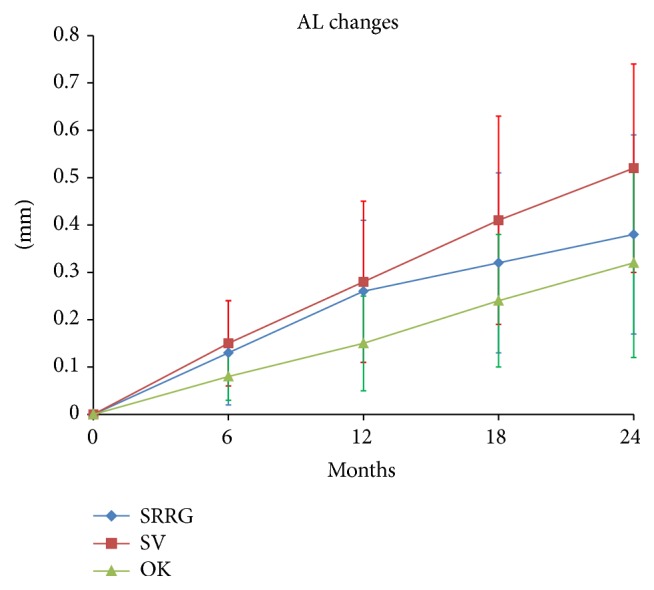
Mean and SD of AL progression (mm).

The AL increased more in the SV group compared to the SRRG and OK groups, respectively; the difference between the SRRG and SV groups was not significant and only approached statistical significance at 2-year visit (*P* = 0.08). The increase in AL in the OK group was significantly lower at all visits compared to the SV group. The mean increases in AL at 2 years were 0.38 ± 0.21 mm, 0.32 ± 0.20 mm, and 0.52 ± 0.22 mm for the SRRG, OK, and SV groups, respectively. In other words, the AL in the SRRG and OK groups increased 27% and 38% less than in the SV group over 2 years. The results are listed in Table 3 and plotted in Figure 5.

The ACD changes at 2 years were 0.16 ± 0.18 mm, 0.11 ± 0.11 mm, and 0.20 ± 0.17 mm for SRRG, OK, and SV groups, which corresponded to 20% and 45% lower increases in the ACD in the SRRG and OK groups compared to the SV group. The differences did not reach significance at any visit.

Crystalline lens thickness did not change in the SRRG group. There was a small increase in the OK group (0.02 ± 0.05 mm) that was significant at 12 and 18 months and 2 years (*P* < 0.05) and a slight decrease in thickness in the SV group (−0.02 ± 0.05 mm).

**Figure 6:**
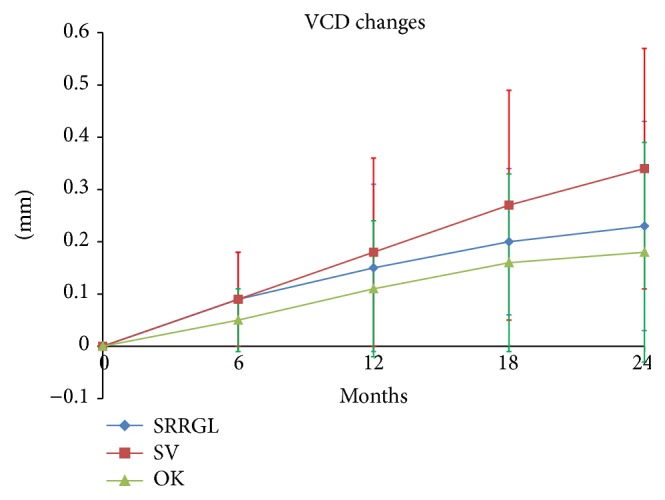
Mean and SD of the VCD changes (mm).

The vitreous chamber increased by 0.23 ± 0.20 mm, 0.18 ± 0.21 mm, and 0.34 ± 0.23 mm in the SRRG, OK, and SV groups, respectively, which corresponded to a 32% lower increase in the SRRG group and a 47% lower increase in the OK group compared to the SV group. The differences obtained at 6, 12, and 18 months and 2 years were not significant (Figure 6). Table 3 shows the results for all parameters.

### 3.5. Slopes of the Progression Lines

The coefficient of determination *r* ^2^ of the regression lines resulted in 0.83, 0.99, and 0.98 for the SRRG, OK, and SV groups, respectively. The AL growth slope was significantly higher in the SV group compared with the SRRG and OK groups (*t*: 147, *P* < 0.05 and *t*: 141, *P* = 0.02 for the SRRG and OK groups versus the SV group, resp.). The SRRG and OK groups did not differ significantly (*t*: 127; *P* = 0.430). The median slopes were 0.016 (95% confidence interval (CI); 0.010–0.017), 0.014 (95% CI, 0.009–0.018), and 0.022 (95% CI, 0.015–0.025), indicating a calculated axial growth of 0.18 mm/year (95% CI, 0.123–0.210), 0.16 mm/year (95% CI, 0.115–0.216), and 0.26 mm/year (95% CI, 0.185–0.305) for the SRRG, OK, and SV groups, respectively.

The VCD change slope was significantly lower for the SRRG and OK groups compared to the SV group (*t*: 161, *P* < 0.01 and *t*: 130, *P* = 0.05 for the SRRG and OK groups versus the SV group, resp.). The SRRG and OK groups did not differ significantly (*t*: 119; *P* = 0.154). The median slopes were 0.009 (95% CI, 0.004–0.010), 0.008 (95% CI, 0.004–0.012), and 0.012 (95% CI, 0.009-0.017). This represents an estimated VCD growth of 0.10 mm/year (95% CI, 0.061–0.136), 0.09 mm/year (95% CI, 0.052–0.151), and 0.15 mm/year (95% CI, 0.115–0.214) for the SRRG, OK, and SV groups, respectively.

Differences in the slope of crystalline lens changes were not significant in any comparison (*t*: 114, *P* = 0.229; *t*: 149, *P* = 0.855 for the SRRG and OK groups compared to the SV group, respectively;, and *t*: 169, *P* = 0.747 for the SRRG group compared to the OK group). The ACD changes were not significant for any comparison: *t*: 182, *P* = 0.898; *t*: 160, *P* = 0.458, for the SRRG and OK groups versus the SV group, respectively; and *t*: 107, *P* = 0.956, for the SRRG group compared with the OK group.

Correlation analysis between axial elongation and baseline refraction showed a weak correlation for either treatment or control groups (*ρ* < 0.300; *P* < 0.05) suggesting that the efficacy of the treatment could not be anticipated as a function of the baseline refraction.

### 3.6. Corneal Parameters

The topographic and pachymetric parameters did not change significantly during the trial between the SRRG and SV groups. However, the mean keratometric reading changed in the SRRG group from 7.60 ± 0.16 mm to 7.56 ± 0.16 mm and pachymetry changed from 534 ± 29 microns to 542 ± 27 microns at 6 months and then returned to 537 ± 32 microns. The eccentricity remained almost unchanged during the study in all groups.

## 4. Discussion

Both the SRRG and OK groups slowed progression of the refractive error by about 43% and 67%, respectively, and they slowed the ocular growth by about 27% and 38%, respectively. Differences between both treatments did not reach significance. While the SV group maintained similar rate of myopia progression throughout the 2 years of the study as before the study, the SRRG and OK groups showed a clear change in the refractive pattern after treatment by slowing the rate of myopic progression in both groups (Figure 5).

The main limitation of the current study was the dropout rate observed over the course of the follow-up. In a recent study that evaluated the differences in myopia progression with a multifocal contact lens, Walline et al. [22] reported the outcomes of 27 of 40 subjects who completed the 2-year study.

Although our dropout rate was high (~42%), it is similar to that in another recent study of DISC lenses [19], and it was similarly distributed in the three groups. Further, the differences between groups were higher than those we initially expected. Therefore, we still maintained a statistical power of 80% to detect differences of 0.75 D and 0.20 mm in refractive changes and axial elongation between the SV group and the other groups with a minimum of 10 subjects in each group. Despite this, further studies with more patients are necessary to confirm the current results.

Our findings supported the myopic progression control effect reported in previous studies of soft multifocal center-distance contact lenses [18, 19, 21]. The mechanism of the effect still needs to be determined with a long-term randomized clinical trial, but it seems clear that peripheral refraction has a potential role. Sankaridurg et al. [18] reported a significant correlation between RPRE measured with multifocal lenses at 30 and 40 degrees in the nasal and temporal retina and progression of myopia. We failed to find a significant correlation between the RPRE (nasal and temporal) and sphere equivalent or AL increase in the SV and SRRG groups probably due the small size sample. However, when all subjects were considered, we found a significant correlation between the RPRE-M and AL change for the first year, and the regression line showed a trend for an inverse relation between a lower refractive increase and more myopic defocus in the nasal and temporal eccentricities. Refractive progression during the year before the trial was correlated with the RPRE at baseline for both retina eccentricities, suggesting that higher degrees of peripheral hyperopia were associated with greater central progression during 1 year before the trial. The baseline sphere also was correlated significantly with the RPRE in the nasal and temporal retina and the amount of baseline sphere was correlated with the amount of progression before the trial, which agreed with the outcome that the nasal retina seems to be related more to myopic progression [25] and relative peripheral hyperopia associated with the amount of central myopia [26]. Nevertheless, in a large population-based cohort study, Mutti et al. [27] showed that the RPRE was not associated with the risk of axial elongation and they concluded that it seemed to exert little consistent effect on the risk of onset of myopic refractive error. Moreover, in a recent study, the baseline relative peripheral hyperopia was not associated with a greater likelihood of becoming myopic or myopia progression [28]. In the current sample, all subjects were myopes with a minimal progression of 0.30 D/year, and the relationship between the RPRE with the refractive correction was correlated with the amount of increase in the AL after 1 year of treatment. More research is needed to elucidate the relationship between the hyperopic RPRE and the likelihood of progression.

Another potential limitation was the measurement of the biometric data done with ultrasonography that requires contact with the eye. This should not be a limitation in the hands of an experienced technician. Further, this instrument allowed us to obtain partial measures of the anterior and posterior dimensions of the eye in addition to the crystalline lens thickness. Biometric data such as the ACD change failed to reach significance, although there was a lower increase by 45% in the OK group and by 20% in the SRRG group compared with the SV group. Changes in crystalline lens thickness were statistically significant, thought small after 2 years in the OK group (0.02 mm). Although this needs further investigation, it might be related with changes in the accommodative function induced by changes in the higher order aberrations of the eye [29]. The vitreous chamber increased less in the SRRG and OK groups compared with the SV group, that is, about 32% and 47%, respectively, but it was not significant at any visit.

We do not know why the refractive change slowed twice as much as the axial increases. A possible limitation of the study was less precise measurement of axial elongation with A-scan ultrasonography compared to partial coherence interferometry. However, ultrasound biometry had been largely used in longitudinal studies of myopia in children as in the CLEERE [30] and COMET [8] studies. Although A-scan ultrasonography is considered sensitive to changes in the AL and VCD equivalent to 0.25 D, it may be a useful technique to assess changes in ocular components in children [31]. In addition, during the study, the measurement methods did not vary and one expert optometrist performed all measurements. Regardless of the fact that we may not discard an excess of pressure when data was acquired, the slope of the changes in VCD also was significant. A recent study over a 3-year period on myopia control with OK also showed discrepancies between the refractive and biometric results. Although the OK group progressed −0.12 D and the control (soft lens) group progressed −1.01 D, the outcomes failed to show AL differences measured by ultrasound between the test and control groups. The authors explained that this finding was due to the high variability between the multiple sites of acquisition [32]. In our case, one expert examiner collected the data using the same procedure and instrument. Furthermore, any bias in the biometric measurements will result in shorter AL measures and narrower ACD. The first will induce a higher slow-down effect closer to the refractive retention effect. The second effect was not observed. Previous studies found a similar difference. For example Walline et al. [22] performed a study of myopic control with a soft multifocal contact lens and obtained changes of 29% and 50% for AL and refractive increases, respectively, and OK results reported by Santodomingo-Rubido et al. [33] showed a 32% reduction effect on AL growth, which is much lower than the normal control effect values reported with OK in Asian children (~50%). Caucasian children have a lower ratio of myopia/AL increase than Asian children. In the CLEERE study [30], the differences in AL growth between emmetropic and myopic children were 0.21 mm/year for the Asian children compared with 0.14 mm/year for the Caucasian children. Moreover, in emmetropic children the AL increased steadily by about 0.10 mm/year compared with the myopic sample at 0.31 mm/year [30]. This means that a 2-year increase in AL for emmetrope is roughly 0.20 mm. Hence, it is illogical to calculate the percentage of myopia control without previously subtracting the emmetropic normal rate of AL growth in all samples. Moreover, seven children were not included in the study due to a low myopia increase and finished the 2-year visit with a refractive change of −0.09 ± 0.27 D and an AL change of 0.19 ± 0.14 mm. In our case, the AL increase after subtraction of the emmetropic children eye grow resulted in 0.18 mm in the SRRG group, 0.12 mm in the OK group and 0.32 mm in the SV group. Those results represent 43% and 62%, which is much more in agreement with the refractive results.

Keratometry and pachymetry did not show any significant changes during the study in any group. In fact, the keratometric variations were considered to readjust the refractive changes from baseline values to avoid confusing results due to anterior corneal changes induced by the soft contact lenses. A limitation of this study was the fact that the uncorrected refractive state of the OK group over the 2 years of the study was impossible to measure due to the induced corneal changes. For this reason, the refractive values in the OK group were evaluated with the lenses on, and to avoid possible lens deformation a final value was adjusted after the power changes of the anterior optical surface of the lens.

Despite the fact that we did not find significant differences between the direct data of the VCD and AL for the SRRG and SV groups, the comparison of the slopes of ocular growth provided clear differences. The slope of the regression line showed the rate of ocular change for each component. This may be a reliable and novel method to analyze ocular changes, and thus it may be useful in future studies of myopia control and ocular growth, leading to a clear understanding of the speed of the ocular changes for each component. The ACD and lens thickness increased similarly among all participants irrespective of their group. The VCD and AL showed similar results, meaning that both variables are well correlated and this eliminates the possibility of a shortened AL by excess pressure, and hence a reduced ACD, at the time of data collection.

OK is currently considered the best optical correction system for myopic control [14, 34]. Studies have shown that the effect on AL growth retention is between 30% and 63% compared to the SV lens or a monofocal contact lens. In this study, we failed to find a decrease in AL growth exceeding 30% for SRRG lens. Both the SRRG and OK groups showed a similar myopic control effect after 2 years, which suggests that the SRRG lens was comparable in this study to OK regarding the AL growing control effect. This effect was consistent during the 2 years of the study. Although the refractive retention effect was better in the OK group with respect to the SRRG group (67% and 43%, resp.), the SRRG lens seems to be a promising optical device that may help to control myopia in children.

## Figures and Tables

**Table 1:** Comparison of demographic and ocular components (mean ± SD) for all subjects initially included in the study, participants who completed the study and those who left the study.

	All			Completed			Discontinued		
	SRRG	OK	SV	SRRG	OK	SV	SRRG	OK	SV
Gender (male; female)	11; 19	16; 13	20; 21	8; 11	10; 8	13; 8	3; 8	6; 5	7; 13
Age	13.07 ± 2.11	12.48 ± 1.50	13.06 ± 2.51	13.34 ± 1.95	12.27 ± 1.76	13.09 ± 2.79	12.21 ± 2.17	13.45 ± 1.07	12.88 ± 2.70
M (D)	−3.76 ± 2.04	−3.44 ± 2.18	−3.11 ± 1.53	−4.46 ± 1.69	−3.51 ± 2.13	−3.61 ± 0.98	−3.62 ± 2.20	−2.86 ± 2.38	−2.03 ± 0.91
Flat *K* (mm)	7.72 ± 0.21	7.86 ± 0.26	7.83 ± 0.23	7.67 ± 0.17	7.79 ± 0.30	7.81 ± 0.20	7.77 ± 0.25	7.85 ± 0.21	7.92 ± 0.28
Steep *K* (mm)	7.57 ± 0.21	7.69 ± 0.27	7.71 ± 0.23	7.53 ± 0.17	7.62 ± 0.29	7.66 ± 0.21	7.62 ± 0.27	7.68 ± 0.21	7.81 ± 0.26
Eccentricity (*e*)	0.48 ± 0.11	0.45 ± 0.08	0.53 ± 0.09	0.47 ± 0.11	0.44 ± 0.08	0.53 ± 0.08	0.49 ± 0.10	0.47 ± 0.08	0.51 ± 0.10
Pachymetry	542 ± 38	537 ± 18	542 ± 28	534 ± 29	538 ± 17	537 ± 24	559 ± 47	547 ± 32	547 ± 47
Anterior chamber	3.84 ± 0.27	3.85 ± 0.23	3.81 ± 0.29	3.84 ± 0.24	3.83 ± 0.19	3.85 ± 0.24	3.86 ± 0.25	3.84 ± 0.24	3.82 ± 0.34
Lens	3.47 ± 0.20	3.46 ± 0.18	3.51 ± 0.18	3.51 ± 0.19	3.53 ± 0.19	3.49 ± 0.19	3.39 ± 0.20	3.41 ± 0.10	3.54 ± 0.13
Vitreous chamber	17.08 ± 0.99	17.46 ± 0.95	17.04 ± 0.74	17.04 ± 0.91	17.23 ± 1.02	17.37 ± 0.83	17.24 ± 1.15	17.48 ± 0.78	16.76 ± 0.79
Axial length	24.38 ± 0.98	24.77 ± 0.89	24.36 ± 0.81	24.38 ± 0.90	24.58 ± 0.95	24.70 ± 0.87	24.46 ± 1.12	24.71 ± 0.71	24.11 ± 0.88

**Table 2:** Mean spherical equivalent increases and SD over 2 years in 6-month intervals. The pre-study annual increase is shown in the first column. Statistically significant results are marked with an asterisk.

	12 months before	6 months	12 months	18 months	24 months
Spherical equivalent changes (D)					
SRRG	−0.76 ± 0.27	−0.06 ± 0.21	−0.28 ± 0.38	−0.39 ± 0.39	−0.56 ± 0.51
OK	−0.75 ± 0.25	−0.16 ± 0.20	−0.26 ± 0.44	−0.32 ± 0.43	−0.32 ± 0.53
SV	−0.62 ± 0.25	−0.27 ± 0.13	−0.53 ± 0.25	−0.80 ± 0.40	−0.98 ± 0.58

Multiple comparisons contrast-*P*					
SV-SRRG	0.660	<0.0001^*∗*^	0.010^*∗*^	0.001^*∗*^	0.010^*∗*^
SV-OK	0.130	0.137	<0.0001^*∗*^	<0.010^*∗*^	0.030^*∗*^
SRRG-OK	0.550	0.092	0.163	0.790	0.965

**Table 3:** Mean and SD of biometric changes (anterior chamber, lens, vitreous chamber, and AL) during 2 years in 6-month intervals. Statistically significant results are marked with an asterisk.

	6 months	12 months	18 months	24 months
*Biometric changes* (mm)				

	Anterior chamber depth (mm)			
SRRG	0.04 ± 0.12	0.13 ± 0.14	0.13 ± 0.16	0.16 ± 0.18
OK	0.03 ± 0.04	0.05 ± 0.08	0.09 ± 0.09	0.11 ± 0.11
SV	0.08 ± 0.12	0.14 ± 0.20	0.17 ± 0.15	0.20 ± 0.17

	Lens thickness (mm)			
SRRG	−0.01 ± 0.05	−0.02 ± 0.06	−0.01 ± 0.04	0.00 ± 0.06
OK	0.00 ± 0.02	0.01 ± 0.04	0.01 ± 0.05	0.02 ± 0.05
SV	−0.02 ± 0.03	−0.04 ± 0.06	−0.03 ± 0.05	−0.02 ± 0.05

	Vitreous chamber depth (mm)			
SRRG	0.09 ± 0.09	0.15 ± 0.16	0.20 ± 0.14	0.23 ± 0.20
OK	0.05 ± 0.06	0.11 ± 0.13	0.16 ± 0.17	0.18 ± 0.21
SV	0.09 ± 0.09	0.18 ± 0.18	0.27 ± 0.22	0.34 ± 0.23

	Axial length (mm)			
SRRG	0.13 ± 0.11	0.26 ± 0.15	0.32 ± 0.19	0.38 ± 0.21
OK	0.08 ± 0.05	0.15 ± 0.10	0.24 ± 0.14	0.32 ± 0.20
SV	0.15 ± 0.09	0.28 ± 0.17	0.41 ± 0.22	0.52 ± 0.22

*Multiple comparisons contrast P for axial length*				

SV-SRRG	0.206	0.800	0.163	0.070
SV-OK	0.02	0.005^*∗*^	0.006^*∗*^	0.008^*∗*^
SRRG-OK	0.214	0.003^*∗*^	0.144	0.346
